# Prognostic role of pretreatment red blood cell distribution width in patients with cancer: A meta-analysis of 49 studies

**DOI:** 10.7150/jca.31598

**Published:** 2019-07-10

**Authors:** Peng-Fei Wang, Si-Ying Song, Hang Guo, Ting-Jian Wang, Ning Liu, Chang-Xiang Yan

**Affiliations:** 1 Department of Neurosurgery, Sanbo Brain Hospital, Capital Medical University, Beijing, China; 2 School of Basic Medical Sciences, Capital Medical University, Beijing, China; 3 Department of Orthopedics, Shanghai Pudong New Area Gongli Hospital, Naval Military Medical University, Shanghai, China

**Keywords:** red blood cell distribution width, malignancies, prognosis, meta-analysis

## Abstract

Red blood cell distribution width (RDW) has been recently demonstrated to be a predictor of inflammation. High pretreatment RDW level is associated with poor survival outcomes in various malignancies, although the results are controversial. We aimed to investigate the prognostic role of RDW. A systematic literature search was performed in MEDLINE and EMBASE till April 2018. Pooled hazard ratios (HRs) were estimated for overall survival (OS) and combined disease-free survival, progression-free survival, and recurrence-free survival (DFS/PFS/RFS). 49 studies with 19,790 individuals were included in the final analysis. High RDW level adversely affected both OS and DFS/PFS/RFS. For solid cancers, colorectal cancer (CRC) had the strongest relationship with poor OS, followed by hepatic cancer (HCC). Negative OS outcomes were also observed in hematological malignancies. Furthermore, patients at either early or advanced stage had inverse relationship between high pretreatment RDW and poor OS. Studies with cut-off values between 13% and 14% had worse HRs for OS and DFS/PFS/RFS than others. Furthermore, region under the curve (ROC) analysis was used widely to define cut-off values and had relatively closer relationship with poorer HRs. In conclusion, our results suggested that elevated pretreatment RDW level could be a negative predictor for cancer prognosis.

## Introduction

Red blood cell distribution width (RDW) is a conventional biomarker for erythrocyte volume variability and an indicator of erythrocyte homeostasis 1. Recent evidence shows that anisocytosis is involved in a variety of human diseases such as cardiovascular diseases 2,3, thrombosis 3, diabetes 4, and cancers 5,6. High RDW level is a negative prognoistic marker for these diseases, and inflammation is the leading mechanism 1.

Inflammation is a key regulator of cancer initiation and progression 7. Recently, RDW, which plays a critical role in inflammatory response, has attracted attention because of the connection between inflammation and cancer. RDW increases in malignant tumors 8,9. Furthermore, higher RDW levels are also significantly associated with advanced stages of cancer and metastasis 10,9.

A mounting body of evidence suggests that elevated RDW level also correlated with poor prognosis for various cancers, which included esophageal cancer 11-15, gastrointestinal tumors 16-18, HCC 19-22, lung cancer 23-26, and hematological malignancies 27-30. However, the prognostic impact of RDW has not been comprehensively investigated because of the inevitable heterogeneity of the samples studied. The aim of the present study was to assess the relationship between RDW and clinical outcomes in patients with cancer.

## Methods

### Search strategy

Our meta-analysis was registered in PROSPERO with the number CRD42018093419. Studies were identified from MEDLINE and EMBASE up to April 2018. Medical subject headings and Emtree headings were searched and combined with the following key-words: “red blood cell distribution width OR RDW” and “prognosis OR prognostic OR survival OR outcome” and “cancer OR tumor OR carcinoma OR neoplasm”. The references of the included articles were also scanned to identify additional studies. Supplementary Table 1 presents the full search strategy.

### Study selection

We included prospective or retrospective studies that assessed RDW level prior to any treatment in patients with proven pathological diagnosis of cancer. Furthermore, eligible studies should provide hazard ratio (HR) with a 95% confidence interval (CI) for clinical outcomes, or enough data to calculate these quantities. We excluded studies based on the time when blood samples were collected; studies were eliminated if they involved patients who received any therapy within two weeks prior to blood donation. Conference abstracts, review articles, case reports, letter, animal studies, or in vitro studies were not eligible for our analysis. Studies with duplicate or overlapping data were also excluded. Two reviewers (PF-W and SY-S) independently performed the study selection and resolved any disagreements via discussion.

### Data extraction

Data from all included studies were extracted by one author (SY-S) and was cross-checked by another author (PF-W). Data were extracted using the name of the first author, year of publication, country, tumor type, clinical/pathological tumor stage, study characteristics (sample size, age, and gender), stage criteria, statistical methods used to calculate the cut-off value for RDW, survival outcomes, and sources of HRs (univariate or multivariate). Furthermore, we calculated the male-to-female gender ratio (M/F gender ratio) to precisely assess the various gender distributions among the included cohorts. The interval of the M/F gender ratio of a balanced composition ranged from one to two; the M/F ratio of a female-dominant composition was less than one, whereas that of male-dominant cohorts was more than two. HRs and 95% CIs were extracted for overall survival (OS), disease-free survival (DFS), progression-free survival (PFS), and recurrence-free survival (RFS). We used the Engauge digitizer to estimate HRs and their 95% CIs if eligible studies provided only Kaplan-Meier curves and we received no response from the investigators after two requests for HRs 31. All disagreements were resolved by consensus.

### Outcomes

We defined OS as the time from the study enrollment to the date of death from any cause or last follow-up. As DFS, PFS, and RFS share similar endpoints, they were analyzed together as one outcome, DFS/PFS/RFS 32-34.

### Statistical analyses

We used STATA version 14.0 (STATA, College Station, TX) in all analyses. Multivariate-adjusted HRs were used when possible, and univariate HRs were included in the meta-analysis if multivariate-adjusted HRs were missing. Pooled estimates with 95% CIs, separately for studies providing OS and DFS/PFS/RFS, were derived using the Mantel-Haenszel method. Further analyses for exploring heterogeneity were comprehensively conducted through subgroup analysis, sensitivity analyses, and meta-regression. Heterogeneity was assessed using the χ^2^ test and expressed as the I^2^ index (25% = low, 50% = medium, 75% = high) 35. A random effects model was used when heterogeneity was > 50%. Alternatively, a fixed effects model was conducted for the meta-analysis. Publication bias was assessed by visual inspection of funnel plots, combined with Egger's test or Begg's test 36,37. Additionally, we applied Duval and Tweede's trim and fill method to estimate corrected effect size after adjustment for publication bias 38. A set of modified predefined criteria was utilized to evaluate the risk of bias in eligible studies 39-41. P-values < 0.05 were considered statistically significant.

## Results

### Study characteristics

**Fig 1:**
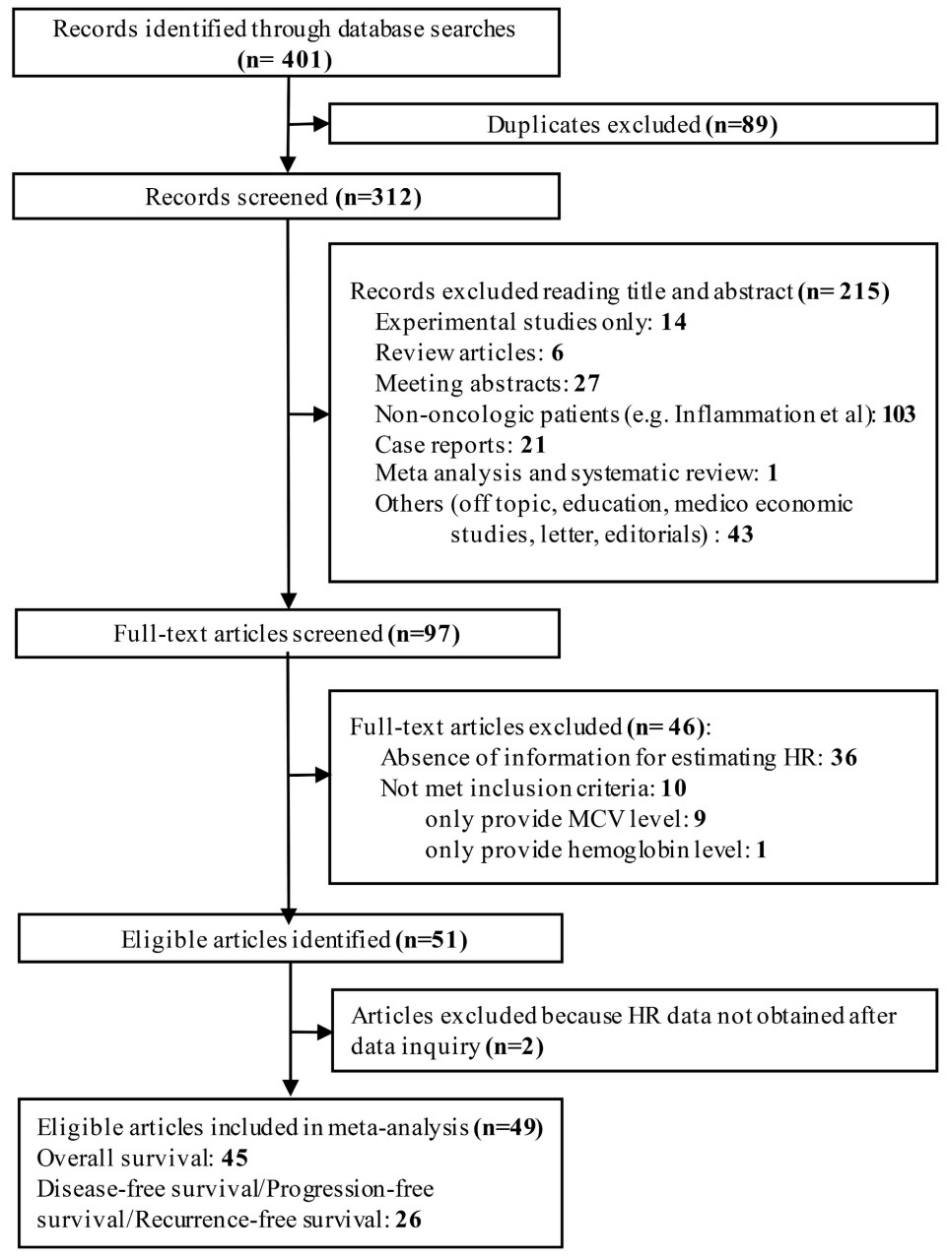
Flow diagram of the study selection process.

Our literature search identified 401 potentially relevant records. Eighty-nine articles were further removed due to duplication. Two-hundred and fifteen studies with irrelevant content were excluded after screening titles and abstracts. Ninety-seven articles were reviewed with full texts. In total, forty-nine studies consisting of 19,790 patients were finally included in our analysis according to the inclusion and exclusion criteria (Fig. 1) 42-47,23,48,11,49,27,28,19,50-52,12,53,24,54,25,55,29,13,26,14,15,20,10,21,56,16,57-62,30,63-66,22,67,17,68,18,69.

The characteristics of the included studies are shown in Table 1. OS and DFS/PFS/RFS were reported in 45 and 26 articles, respectively. Sixteen different solid cancer types and five different hematological malignancies were investigated in the eligible studies. For solid tumors, the most frequently evaluated cancer was upper gastrointestinal cancer (UGI) (including patients with pancreatic, esophageal, and gastric cancer) (n = 8), followed by hepatic cancer (HCC) (n = 4), non-small cell lung cancer (NSCLC) (n = 4), colorectal cancer (CRC) (n = 3), breast cancer (n = 3), and glioma (n = 3). Multiple myeloma (MM) (n = 5) and diffuse large B-cell lymphoma (DLBCL) (n = 2) were the most-studied diseases among hematological malignancies. A large number of studies (90%) enrolled patients with mixed-stage, whereas only a few studies specifically investigated patients with early- (10%) and advanced-stage (12%) disease. Five different methods for defining cut-off values were observed in the included studies. Region under the curve (ROC) analysis was used most frequently (n = 23), followed by the upper limit of reference range (n = 12) and empirical values based on previous studies (n = 6). With respect to cut-off values, most studies (94%) selected coefficient of variation (CV) to evaluate RDW, whereas others used standard deviation (SD). The cut-off values ranged from 12.20% to 20.00%. However, thirty-six studies (80%) applied cut-off values in the range of 13-15%. Furthermore, we evaluated the demographic characteristics among the cohorts, such as age, gender, and country of origin. Twenty-two studies (52%) enrolled elderly population, the median or mean age of whom was > 60 years. The number of cohorts with balanced gender composition (n = 22) was nearly equal to that of cohorts with female or male dominant composition (n = 24). Sixty-three percent cohorts were originally from Asian countries, whereas the others were from Western countries. In our assessment of study quality, nine studies had quality scores ≤ 7, and the remaining 40 studies had scores > 7 (Supplementary Table 3).

### Overall survival

**Fig 2:**
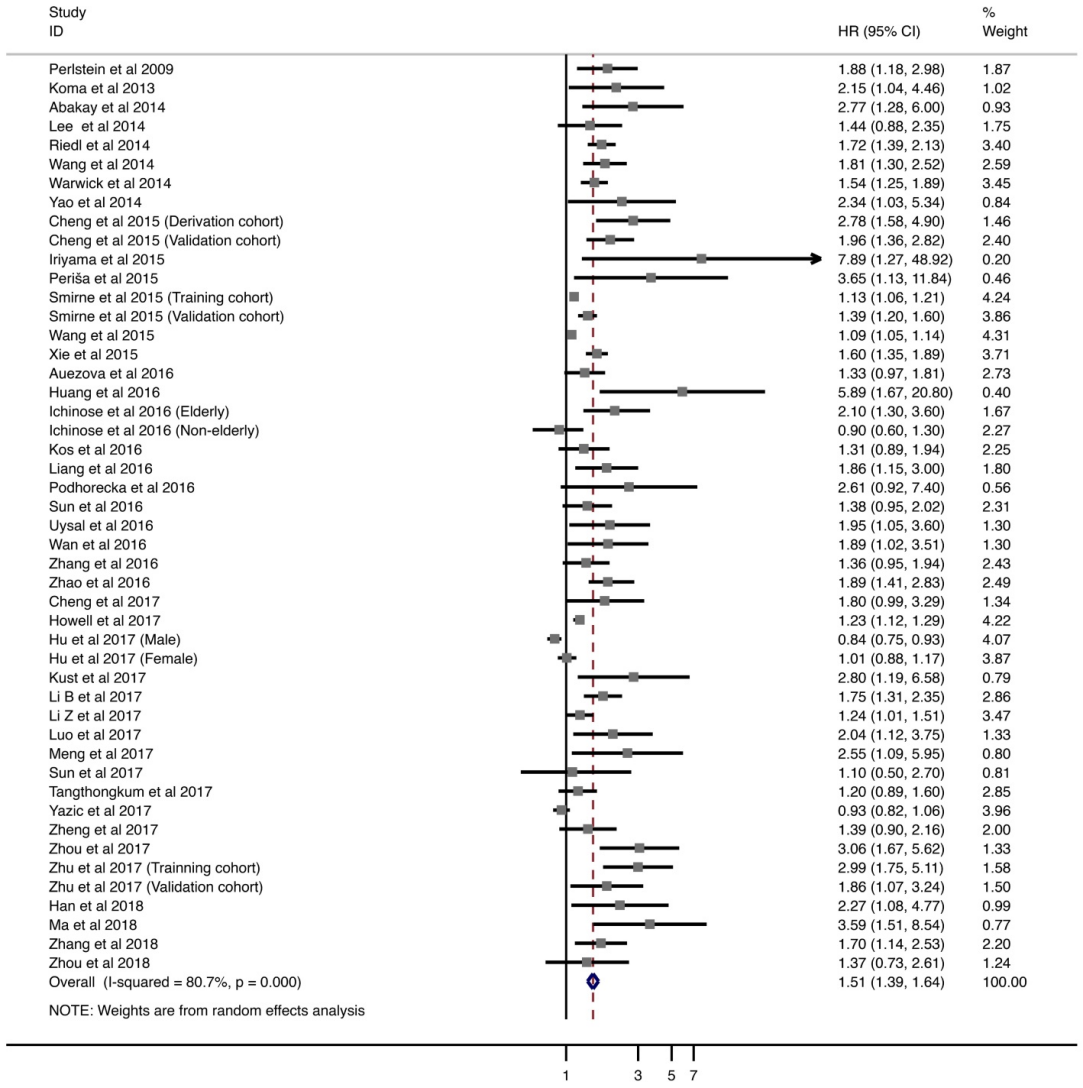
Meta-analysis of the association between RDW and OS in patients. Results are presented as individual and pooled hazard ratios (HRs) with 95% confidence intervals (CIs).

Forty-five studies with 18,767 patients were analyzed for OS. The pooled HRs of higher pretreatment RDW level was 1.508 (95% CI = 1.387-1.639; Fig. 2). Next, we performed comprehensive analysis to explore the high heterogeneity, including subgroup analyses, sensitivity analysis, and meta-regression.

Table 2 shows the subgroup analysis of the included studies, based on eight factors, including tumor type, tumor stage, age, gender distribution, country of origin, cut-off value, method of defining the cut-off value, and HR calculation. In solid tumors, CRC had the strongest relationship with poor OS (HR = 1.932; 95% CI = 1.397-2.673), followed by HCC (HR = 1.430; 95% CI = 1.232-1.660) and NSCLC (HR = 1.440; 95% CI = 1.103-1.880). However, UGI cancer and breast cancer with elevated RDW were not associated with worse OS (UGI cancer: HR = 1.091; 95% CI = 0.925-1.286. Breast cancer: HR = 2.092, 95% CI = 0.833-5-255). For hematological malignancies, negative OS outcomes were observed in MM and DLBCL (MM: HR = 1.692; 95% CI = 1.256-2.281. DLBCL: HR = 3.178, 95% CI = 1.853-5.450). In addition, patients in either early or advanced stage showed adverse relationship between increased pretreatment RDW and poor OS. Furthermore, combined HR remained significant in subgroups stratified by demographic factors, including age, gender, and country of origin. Studies with cut-off values between 13% and 14% had worse HR than others. However, considerable variety was present in the methodologies used for defining cut-off values. ROC analysis was the most widely used method and had relatively closer relationship with poorer HRs. Finally, studies using univariate (HR = 1.525; 95% CI = 1.380-1.686) and multivariate analyses (HR = 1.477; 95% CI = 1.342-1.626) showed that higher RDW levels were associated worse OS.

In sensitivity analysis under “one study removed” model, the pooled HRs for OS were significantly affected by exclusion of Wang et al. (Supplementary Table 4). In addition, meta-regression did not demonstrate any potential source of heterogeneity (Supplementary Table 5).

### DFS/PFS/RFS

**Fig 3:**
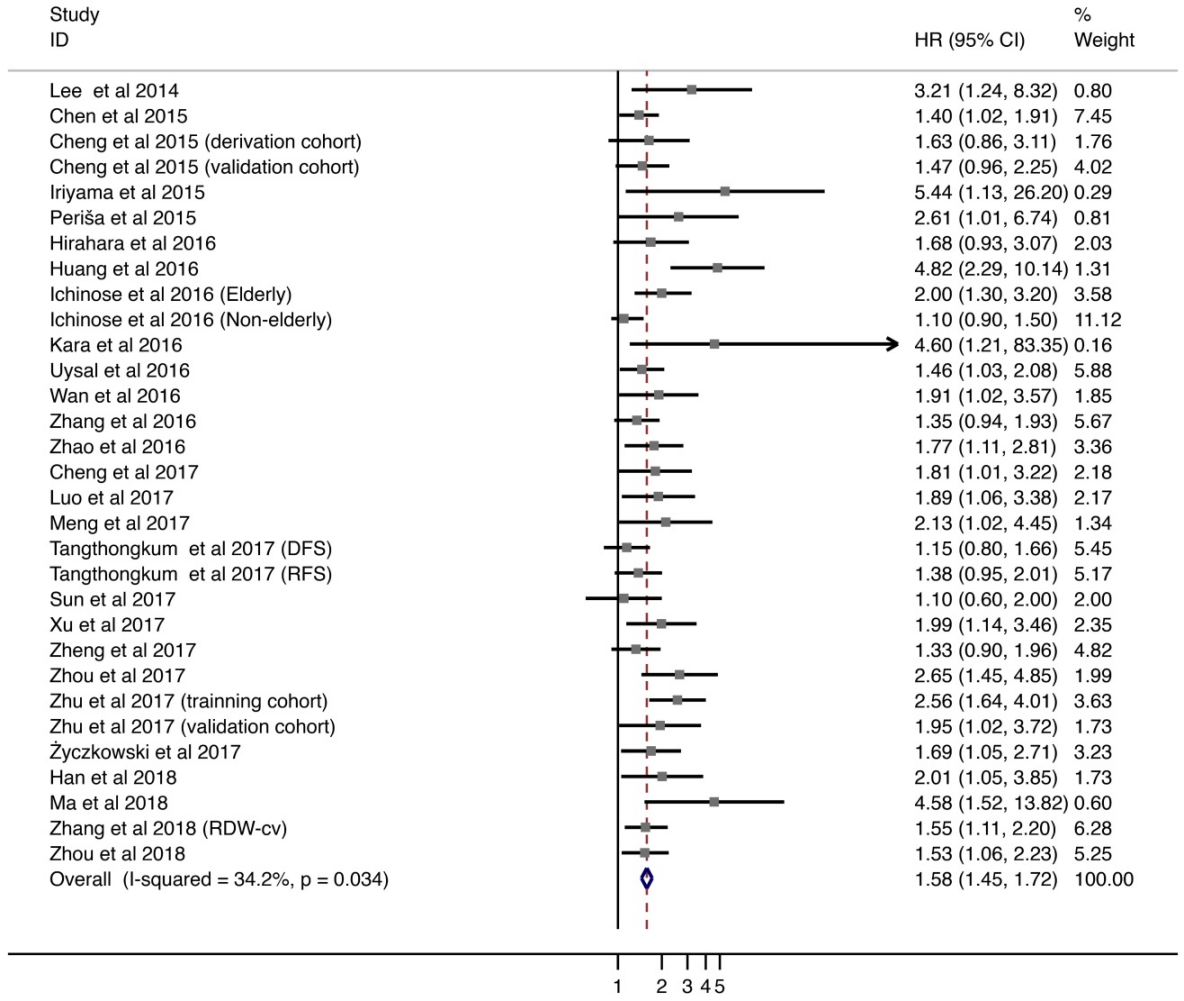
Meta-analysis of the association between RDW and DFS/PFS/RFS in patients. Results are presented as individual and pooled hazard ratios (HRs) with 95% confidence intervals (CIs).

Twenty-six studies with 7,350 patients provided HRs and 95% CIs for DFS/PFS/RFS. Overall, elevated pretreatment RDW level were associated with worse DFS/PFS/RFS (HR = 1.576; 95% CI = 1.447-1.716; Fig. 3). Subgroup analyses were performed by stratification based on tumor type, tumor stage, age, gender distribution, country of origin, cut-off value, method of defining the cut-off value, and HR calculation (Supplementary Table 2). Higher levels of RDW were associated with shorter DFS/PFS/RFS in patients with HCC (HR = 2.104, 95% CI = 1.577-2.807), CRC (HR = 1.636; 95% CI = 1.211-2.211), and hematological malignancies (HR = 2.077; 95% CI = 1.644-2.625).

Overall, HRs remained significant in subgroups stratified by demographic factors, including age, gender, and country of origin. Furthermore, associations between higher RDW levels and worse DFS/PFS/RFS were also observed with cut-off values > 13% and < 14% (HR = 1.818; 95% CI = 1.474-2.243). Studies which utilized ROC analysis to define cut-off values showed comparatively worse HRs (HR = 1.770; 95% CI = 1.536-2.040). Finally, both univariate and multivariate analyses for HR calculation indicated poor DFS/PFS/RFS outcomes.

### Publication bias

We observed evidence of publication bias in studies provided on OS (n = 45) and DFS/PFS/RFS (n = 26) by visual inspection of the funnel plot (Supplementary Fig. 1), which was further confirmed by Egger's tests (P < 0.001) (Supplementary Fig. 2). The trim and fill method was applied to address these problems. Intriguingly, pooled adjusted HRs of OS and DFS/PFS/RFS subsets were consistent with our primary analysis (Supplementary Table 6 and Supplementary Fig. 3).

## Discussion

RDW is an easily acquired, non-invasive, and inexpensive maker, which can be used routinely for clinical purpose. This is the first meta-analysis to comprehensively evaluate the prognostic role of RDW in cancers. High RDW level was correlated with unfavorable clinical outcomes in most tumor types and stages. The prognostic value of RDW was also independent of patient age, gender, or region.

Gradual increase in RDW with age has been reported in healthy people 1. However, association between gender and RDW is still unclear. Certain studies indicated that RDW was slightly higher in females 70,71, whereas others observed no significant gender-based difference in RDW values 72,73. Hence, an age- and gender-stratified subgroup analysis was performed. Poor survival outcome was associated with higher RDW in elder or younger patients with cancer. Similarly, both females and males with high RDW levels exhibited poor survival. These results showed that RDW can predict survival independent of age and gender. The cut-off value of 14.6% is conventionally used for anemia 74. However, the lack of unified RDW cutoff values for cancer survival prediction was a matter of concern 73.

Majority of the studies used ROC analysis to define cut-off values, which ranged from 12.20% to 20.00%. However, 36 studies (80%) applied cut-off values between 13% and 15%. We observed that cut-off values defined by ROC curves were more likely to predict poor clinical outcomes. Furthermore, subgroups with cut-off values between 13% and 14% were mostly negatively associated with poor OS and DFS/PFS/RFS. We conclude that more studies are required to determine uniform cut-off values in specific cancer types.

The mechanisms underlying the prognostic impact of RDW on cancers were due to inflammation 75, poor nutritional status 76, and oxidative stress 77. First, it is well-known that malignant tumors are accompanied by systemic inflammatory response 76. RDW was identified as an inflammatory marker in patients with cancer due to its positive association with widely used plasma inflammatory biomarkers such as C-reactive protein (CRP) 43,28,14, erythrocyte sedimentation rate (ESR) 60,47, and interleukin (IL)-6 78 levels. Elevated RDW level reflected the presence of immature juvenile red blood cells in the periphery. Various cytokines affect erythropoiesis via erythropoietin (EPO) production, inhibition of erythroid progenitors, and reduction in iron release. Previous *in vitro and in vivo* studies have demonstrated that EPO production was inhibited by inflammatory cytokines 79-81 such as IL-6, interferon-gamma (IFN-γ), IL-1β, and tumor necrosis factor-alpha (TNF-α). In addition, IL-1α and IL-1β play important roles in suppression of erythroid progenitors 82. Hepcidin, a regulator of iron metabolism, is increasingly expressed when plasma IL-6 level is elevated 83,84, which results in iron deficiency and anemia 80. In sum, it is plausible to hypothesize that RDW can reflect inflammatory status in cancer. Second, malnutrition is another hallmark of cancer because of reduction in appetite and weight. This results in deficiency of various minerals and vitamins such as iron, folate and vitamin B12, which consequently contribute to the increase in RDW 85,42. Numerous studies have also shown that low albumin level is associated with increased RDW level in cancer patients 24,60,30,69, which also indicated the relationship between high RDW level and poor nutritional status in patients with cancer. Third, oxidative stress was recognized as a negative factor leading to significant variation in erythrocyte size. Free reactive oxygen species (ROS) can damage protein, lipids, and DNA, which may reduce RBC survival 86. Taken together, high RDW level is well-suited to reflect both chronic ongoing inflammation and poor nutritional status in patients with cancer.

Among solid tumors, CRC and HCC showed relatively strong association between RDW level and negative prognosis. This significant association in CRC may be attributed to chronic inflammatory status and cancer-associated anemia. CRC can develop from inflammatory bowel diseases and inflamed polyps 87-89. Thus, inflammation plays a crucial role in colorectal carcinogenesis 90. In addition, chronic blood loss is a common symptom of CRC, which can lead to iron deficiency, anemia, and subsequent rise in RDW values. HCC is one of the most important inflammation-associated cancers 91; it is closely associated with chronic inflammation and fibrosis, which is known as hepatic inflammation-fibrosis-cancer (IFC) axis. IL-6 and TNF-α expression was elevated and erythrocyte maturation was suppressed in patients with HCC 92. Furthermore, within the diseased liver, free radicals such as ROS and nitrogen species (NO) were generated by the cells of the hepatic immune system, including recruited neutrophils, monocytes, and Kupffer Cells 92. In sum, elevated RDW was negatively associated with the prognosis of certain cancer types, which encompassed multiple pathways affecting erythropoiesis.

In our meta-analysis, pretreatment RDW was identified as a robust predictor of cancer prognosis. However, there are several limitations. First, there was considerable heterogeneity when HRs for OS outcomes were pooled. However, subgroup analysis showed that various methodologies for defining cut-off values may be a major cause of heterogeneity. The robustness of our results was further confirmed by sensitivity analysis and meta-regression, which did not significantly alter the pooled effect size for OS. Second, we observed that some studies evaluated the relationship between delta RDW level 17,27,16 or delta MCV level 93-96 and cancer prognosis after the patients had undergone certain therapies. However, we focused on the prognostic role of absolute value of pretreatment RDW level in this analysis as delta RDW level may be dependent on many cofactors such as therapies and types of cancer. Finally, although pretreatment RDW level can reflect both inflammatory and nutritional status, it would be more convincible if combined with other potential predictors, such as neutrophil to lymphocyte ratio (NLR) and prognostic nutritional index (PNI). More studies are required for building a new prognostic and comprehensive model for predicting survival outcomes in patients with cancer.

## Conclusions

Pretreatment RDW level is a potential predictor of cancer prognosis, independent of most tumor type and stage and patient age and gender. Optimal RDW cut-off values can be defined by ROC analysis. Cut-off values between 13% and 14% were negatively associated with poor survival outcomes. Uniform cut-off values for specific cancer types are required for further evaluation in future.

## Supplementary Material

Supplementary figures and tables.

## Figures and Tables

**Table 1:** Main characteristics of 49 eligible studies included in the meta-analysis.

Study, Year	Country	Tumor type	Study design	Stage	Criteria	Sample size	Age^a^	Gender (Female/male)	Definition of cut-offs	Cut-offs value	Outcome measures	HRs source	variables
Perlstein et al 2009	USA	NR	prospective	NR	NR	NR	NR	NR	4th quartile	14.35%	OS	UV	
Koma et al 2013	Japan	Lung cancer	retrospective	I-IV	UICC-7	332	71.5 (38-94)	109/223	Upper limit	15.00%	OS	UV; MV	RDW; Stage; ECOG PS; Other diseases; Treatment; Albumin; CRP
Abakay et al 2014	Turkey	Malignant mesothelioma	retrospective	NR	NR	152	58.2 ± 11.9	65/90	Arbitrary^c^	20.00%	OS	MV	RDW; Histopathological subtype; NLR
Lee et al 2014	Korea	MM	retrospective	I-III	ISS	146	61 (32-83)	55/91	Upper limit	14.50%	OS; PFS	UV; MV	RDW; Age at diagnosis; ECOG; Cytogenetic risk; B2MG; Albumin; LDH; Hemoglobin; Calcium; Induction with novel agents; ASCT
Riedl et al 2014	Austria	Multiple malignancies^b^	prospective	Localized; Distant metastasis; Not classifiable	NR	1840	62 (52-68)	843/997	Upper limit; 4th quartile	16%; 14.6%	OS	UV; MV	RDW; Age; Sex
Wang et al 2014	China	RCC	retrospective	I-IV	AJCC-7	316	56.83 ± 11.68	108/210	ROC	12.85%	OS	MV	RDW; Smoking; Hemoglobin; MCV; Platelet; WBC; Albumin; ESR
Warwick et al 2014	UK	NSCLC	retrospective	T1-3; N0-1	AJCC-7	917	67.21 (17-90)	440/477	4th quartile	15.30%	OS	MV	RDW; Age; Alcohol intake; Emphysema; Squamous carcinoma; predicted postoperative FEV1; T stage I; T stage III; N stage I
Yao et al 2014	China	Breast cancer	retrospective	Tis-T3; N0-3	NR	608	52.4 ± 10.8	608/0	ROC	13.45%	OS	MV	RDW; Node stage; Molecular subtype; NLR
Chen et al 2015	China	ESCC	retrospective	T1-4; N0-3	NR	277	NR	37/240	Mean	14.50%	CSS	MV	RDW; Tumor length; Vessel invasion; Differentiation; T stage; N stage
Cheng et al 2015	Taiwan	UTUC	retrospective	Tis-T4; N0-+	AJCC-6	420	68 ± 10.3	116/79	Within central 80 % distribution.	14.00%	OS; CSS	UV; MV	RDW; T stage; LN metastasis; Tumor grade; Adjuvant chemotherapy; WBC; NLR
Iriyama et al 2015	Japan	CML	retrospective	NR	NR	84	51 (22-85)	30/54	Arbitrary^c^	15.00%	OS; EFS	UV	
Periša et al 2015	Croatia	DLBL	retrospective	I-IV	Ann Arbor	81	64.0 (52.5-72.5)	52/29	ROC	15.00%	OS; EFS	MV	RDW; Age; Sex; IPI; LDH; Clinical stage AA; ECOG PS
Smirne et al 2015	Italy	HCC	retrospective	A-D	BCLC	314	Training cohort 70 (62-77); Validation cohort 67 (59-74)	Training cohort 52/156; Validation cohort 26/80	Upper limit	14.60%	OS	MV	RDW; Age at diagnosis; BCLC stage; Child-Pugh-Turcotte score; tumor size; serum AFP
Wang et al 2015	USA	Breast cancer	retrospective	I-IV	AJCC-6	1816	Black 57.26 ± 13.99; White 60.05 ± 13.43	1816/0	NR	14.50%	OS	MV	RDW; Age; Year of diagnosis; Ethnicity; Smoking status, Drinking status; Stage; Grade; Estrogen receptor status; progesterone receptor status
Xie et al 2015	USA	SCLC	prospective	Extensive; Limited	NR	938	65.4 ± 11.0	438/500	Upper limit	15.00%	OS	UV; MV	RDW; NLR; PLR; Age at diagnosis; Gender; ECOG performance status; Chest radiation; Chemotherapy; Liver metastases; Numbers of metastatic sites
Auezova et al 2016	Kazakhstan	Gliomas	retrospective	Grade I-IV	WHO 2007	178	41.58 ± 1.04	85/93	ROC	13.95%	OS	UV	
Hirahara et al 2016	Japan	ESCC	retrospective	I-III	AJCC-7	144	NR	15/129	Upper limit	50fL	CSS	UV; MV	RDW; Stage; Tumor size; Operation time
Huang et al 2016	China	Breast cancer	retrospective	I-III	AJCC-6	203	37 (24-40)	203/0	ROC	13.75%	OS; DFS	MV	RDW; PVI present; PR positive; Stage
Ichinose et al 2016	Japan	NSCLC	retrospective	T1-4; N0-2	UICC-7	992	NR	NR	Median	13.80%	OS; DFS	MV	RDW; Gender; T factor; N factor; Sub-lobar resection; CEA; NLR; Albumin; Smoking
Kara et al 2016	Turkey	Laryngeal carcinoma	retrospective	T1-4; N0-2; M0	AJCC-7	103	65.01 ± 9.01	NR	ROC	14.05%	OS	MV	RDW; Tumor stage
Kos et al 2016	Turkey	NSCLC	retrospective	I-IV	UICC-7	146	56.5 (26-83)	15/131	Median; ROC; Upper limit; Arbitrary^c^	14%; 14.2%; 14.5%; 15%	OS	UV	
Liang et al 2016	China	Glioblastoma	retrospective	NR	NR	109	54 (19-85)	42/67	ROC	14.10%	OS	MV	RDW; Age; Tumor location; Extent of resection; Adjuvant radio/chemotherapy; MCV; MCHC
Podhorecka et al 2016	Poland	CLL	retrospective	0-IV	Rai	66	63 (38-85)	25/38	Upper limit	14.50%	OS	UV	
Sun et al 2016	China	ESCC	retrospective	I-III	AJCC-6	362	Median 58; Mean 57.96	94/268	ROC	13.60%	OS; DFS	UV	
Uysal et al 2016	Turkey	NSCLC	retrospective	IA-IIIA	NR	249	60.8 ± 9.1	41/208	Upper limit	14.60%	OS; DFS	UV	
Wan et al 2016	China	ESCC	retrospective	I-III	AJCC/ UICC-7	179	63.0 (42-77)	29/150	Upper limit	15.00%	OS; DFS	MV	RDW; Stage (III vs. I&II); Node metastasis status; Tumor length; WBC; Albumin; CRP; NLR
Zhang et al 2016	China	ESCC	retrospective	I-III	AJCC-7	468	59.5 ± 9.0; 60 (36-81)	92/376	ROC	12.20%	OS; DFS	MV	RDW; Age; N metastasis; Adjuvant radio/chemotherapy; Smoking; Maximum tumor diameter; MCV; CA19-9; NLR; PLR; COP-MPV
Zhao et al 2016	China	HCC	retrospective	I-IV	NR	106	52 (22-75)	13/93	Upper limit	14.50%	OS; DFS	MV; UV	RDW; TNM stage; Tumor size; Tumor number; Vascular invasion
Cheng et al 2017	China	GC	retrospective	I-IV	AJCC-7	227	NR	51/176	Median	13.00%	OS; DFS	UV	
Howell et al 2017	Japan, Italy and UK	HCC	prospective	A-D	BCLC; CLIP scores	442	69.92 ± 10.06	96/346	NR	NR	OS	MV	Treatment-naïve HCC; NLR; CLIP score; Diarrhea on sorafenib; RDW
Hu et al 2017	China	ESCC	retrospective	I-III	AJCC/ UICC-7	2396	Male 55.98 ± 9.81; Female 57.93 ± 9.41	574/1822	NR	NR	OS	MV	Age, body mass index, smoking, drinking, family history of cancer, systolic blood pressure, fasting blood glucose, TNM stage, tumor embolus and tumor size
Kust et al 2017	Croatia	CRC	retrospective	I-IV	AJCC-7	90	66.8 ± 9.7	37/53	ROC	14.00%	OS	MV	RDW; Age; Gender; AJCC stage; NLR
Li B et al 2017	China	Hilar cholangiocarcinoma	retrospective	I-IV	AJCC-7	292	60 (20-78)	131/161	ROC	14.95%	OS	MV	RDW; Histologic grade; T stage; N stage; AJCC stage; Portal vein invasion; Hepatic artery invasion
Li Z et al 2017	USA	Epithelial ovarian cancer	retrospective	I-IV	NR	654	63 (28-93)	654/0	ROC	14.15%	OS	MV	RDW; NLR; PLR; MLR; Combined RDW+NLR; Stage; Origin of cancer; Age; Histology; Grade; Residual disease
Luo et al 2017	China	Nasal-type, extranodal natural killer/T-cell lymphoma	retrospective	I-IV	Ann Arbor	191	44 (15-86)	57/134	ROC	46.2 fL	OS; PFS	MV	RDW; Local invasiveness; Hemoglobin
Meng et al 2017	China	MM	retrospective	I-III	DSS	166	61.6 ± 10.8	78/88	Arbitrary^c^	14.00%	OS; PFS	UV	
Sun et al 2017	China	Prostate cancer	retrospective	NR	NR	171	68.5 ± 8.4	0/171	ROC	12.90%	OS	UV	
Tangthongkum et al 2017	Thailand	Oral cancer	retrospective	I-IV	AJCC-7	374	60 (21-92)	133/241	Arbitrary^c^	14.05%	OS; DFS; RFS	UV; MV	RDW; Stage; PLR
Wang et al 2017	China	MM	retrospective	I-III	ISS	196	65 (33-82)	86/110	ROC	18.05%	OS	MV	RDW; Age; gender; Albumin; Lactate dehydrogenase; Creatinine
Xu et al 2017	China	Glioma	retrospective	Low grade; High grade	WHO 2007	168	44.1 ± 14.6	168/0	NR	13.20%	PFS	UV	
Yazic et al 2017	Turkey	GC	retrospective	I-III	AJCC/ UICC-7	173	61.7 ± 12	62/110	Mean	16.00%	OS	MV	RDW; Gender; Age; Tumor diameter; Vascular invasion; PNI; Metastatic LN; PRBC; Complication; T1; PDW; MCV
Zheng et al 2017	China	Cervical cancer	retrospective	IA1-IIA2	FIGO	800	49.5 ± 10.7	800/0	ROC	12.70%	OS; DFS	UV	
Zhou et al 2017	China	DLBL	retrospective	I-IV	Ann Arbor	161	59.1±11.4	70/91	ROC	14.10%	OS; PFS	MV	
Zhu et al 2017	China	HCC	retrospective	I-III	NR	316	52.2 (22.0-80.0)	Training cohort 26/159; Validation cohort 20/111	ROC	13.25%	OS; DFS	MV; UV	RDW; FIB-4; NLR; PLR; Liver cirrhosis; Tumor size; Tumor capsule; Tumor thrombus; TNM stage
Życzkowski et al 2017	Poland	RCC	retrospective	I-IV	AJCC-7	434	62.0 (54.0-69.0)	203/231	ROC	13.90%	CSS	MV	RDW; Age; Gender; T stage; Distant metastases; Nephrectomy; Tumor necrosis; Grading
Han et al 2018	China	CRC	retrospective	I-IV	NR	128	NR	167/73	ROC	13.45%	OS; DFS	UV; MV	RDW; Differentiation; CA19‐9
Ma et al 2018	China	MM	retrospective	I-III	ISS; DSS	78	60.7 (43-81)	31/47	ROC	15.50%	OS; PFS	UV	RDW; B symptoms; IPI; ECOG PS; LDH; Stage; Bone marrow involvement; Extranodal sites of disease; Hemoglobin
Zhang et al 2018	China	Rectal cancer	retrospective	I-III	AJCC-7	625	NR	241/384	ROC	RDW-cv 14.1%; RDW-sd 48.2fL	OS; DFS	MV	RDW; Tumor location; Tumor size; Differentiation; TNM; Vascular invasion; Perineural invasion
Zhou et al 2018	China	MM	retrospective	I-III	ISS	162	61 (40-87)	75/87	Upper limit	14.00%	OS; PFS	UV	

**Abbreviations:** GC = gastric cancer; ESCC = esophageal squamous cell carcinoma; CRC = colorectal carcinoma; HCC = hepatocellular carcinoma; NSCLC = non-small cell lung cancer; SCLC = small cell lung cancer; RCC = renal cell cancer; UTUC = Upper tract urothelial carcinoma; MM = multiple myeloma; chronic lymphocytic leukemia = CLL; CML = Chronic Myeloid Leukemia; DLBL = diffuse large B-cell lymphomas; AJCC = The American Joint Committee on Cancer; BCLC = Barcelona Clinic Liver Cancer guidelines; UICC = International Union Against Cancer; DSS = Durie and Salmon staging system; ISS = International Staging System; OS = overall survival; PFS = progression free survival; RFS = recurrence free survival; DFS = disease free survival; event-free survival = EFS; MV = multivariate; UV = univariate; RDW-CV = red blood cell distribution width coefficient of variation; RDW-SD = red blood cell distribution width standard deviation; NR = not reported a. Age reported as either mean ± standard deviation or median (range), if not otherwise specified. b. Multiple malignancies include brain, breast, lung, upper or lower gastrointestinal tract, pancreas, kidney, prostate or gynecological system; sarcoma and hematologic malignancies (lymphoma, multiple myeloma) c. Studies defined cut-offs value based on previous studies.

**Table 2:** Subgroup analyses of the associations between RDW and OS in cancer.

Stratified analyses	No. of patients	No. of studies	Model	Pooled HR (95%CI)	P value	P_D_ value	Heterogeneity	
							I^2^	P_H_ value
**Tumor type**						<0.001		
Hematologic malignancies	1979	10	fixed	2.046 (1.623-2.580)	<0.001		21.2%	0.248
MM	748	5	fixed	1.692 (1.256-2.281)	0.001		18.8%	0.295
DLBCL	881	2	fixed	3.178 (1.853-5.450)	<0.001		0.0%	0.793
UGI cancer	3805	6	random	1.091 (0.925-1.286)	0.303		73.4%	0.001
HCC	1510	5	random	1.430 (1.232-1.660)	<0.001		79.9%	<0.001
NSCLC	2304	4	random	1.440 (1.103-1.880)	0.007		57.2%	0.053
Breast cancer	2627	3	random	2.092 (0.833-5.255)	0.116		80.3%	0.006
Colorectal carcinoma	843	3	fixed	1.932 (1.397-2.673)	<0.001		0.0%	0.521
Gliomas	287	2	fixed	1.466 (1.129-1.904)	<0.001		23.9%	0.252
UTUC	420	1*	fixed	2.172 (1.599-2.949)	<0.001		3.5%	0.309
**Stage**						<0.001		
Mix stage	16786	33	random	1.494 (1.372-1.626)	<0.001		80.5%	<0.001
Early stage	1545	5	fixed	1.690 (1.180-2.422)	0.004		41.0%	0.148
Advanced Stage	1416	6	random	1.717 (1.235-2.386)	0.001		57.7%	0.038
**Age**						<0.001		
≤60	7979	19	random	1.590 (1.321-1.914)	<0.001		82.6%	<0.001
>60	7992	22	random	1.515 (1.351-1.699)	<0.001		75.7%	<0.001
**Gender distribution**						<0.001		
Female dominant	5059	9	random	1.401 (1.153-1.703)	0.001		74.9%	0.001
Balanced	6418	21	random	1.696 (1.441-1.997)	<0.001		74.8%	<0.001
Male dominant	5325	14	random	1.413 (1.232-1.620)	<0.001		81.6%	<0.001
**Country**						<0.001		
Eastern	10608	28	random	1.716 (1.458-2.020)	<0.001		79.8%	<0.001
Western	8180	17	random	1.316 (1.203-1.439)	<0.001		80.9%	<0.001
**Cut-off value**						<0.001		
>15%	3356	6	random	1.608 (1.107-2.335)	0.013		89.5%	<0.001
>14% and ≤ 15%	7911	21	random	1.510 (1.351-1.688)	<0.001		79.2%	<0.001
>13% and ≤ 14%	3409	11	random	1.869 (1.493-2.340)	<0.001		57.5%	0.004
≤13%	1982	5	fixed	1.534 (1.262-1.865)	<0.001		0.0%	0.655
**Definition of cut-off value**						<0.001		
ROC curve analysis	6276	22	fixed	1.569 (1.434-1.718)	<0.001		42.6%	0.015
Upper limit	3558	11	random	1.504 (1.296-1.746)	<0.001		70.8%	0.000
Median	2357	3	random	1.400 (0.961-2.040)	0.080		62.4%	0.046
4th quartile	2757	3	random	1.647 (1.430-1.897)	<0.001		0.0%	0.645
Arbitrary^#^	922	5	random	1.682 (1.073-2.638)	0.023		63.2%	0.028
**HR calculation^‡^**						<0.001		
Multivariate	13572	28	random	1.477 (1.342-1.626)	<0.001		83.9%	<0.001
Univariate	4275	17	fixed	1.525 (1.380-1.686)	<0.001		8.5%	0.355

**Abbreviations:** MM = Multiple Myeloma; DLBCL = Diffuse large B-cell lymphoma; UGI cancer = upper gastrointestinal tract (UGI) cancers (including esophagus cancer, gastric cancer, and small intestine cancer); HCC = hepatocellular carcinoma; NSCLC = non-small cell lung cancer; UTUC = upper tract urothelial carcinoma; OS = overall survival; HR = hazard ratio; CI = confidence interval; P_D_ = P for subgroup difference; P_H_ = P for heterogeneity. *: Cheng et al 2015 separately evaluated the survival outcome in two cohorts, which were derivation cohort and validation cohort. #: Definition of cut-offs value of RDW was based on previous study. ‡: HRs were extracted from multivariate cox proportional hazards models, univariate cox proportional hazards models or survival curve analysis.
