# Supplementary material for: Prognostic role of pretreatment red blood cell distribution width in patients with cancer: A meta-analysis of 49 studies
Source: J Cancer. 2019 Jul 10;10(18):4305–17. doi: 10.7150/jca.31598 (PMC6691718; doi:10.7150/jca.31598)
Supplement: Supplementary file 1 — Supplementary figures and tables. [file jcav10p4305s1.pdf]

Supplementary figure

Fig.1A

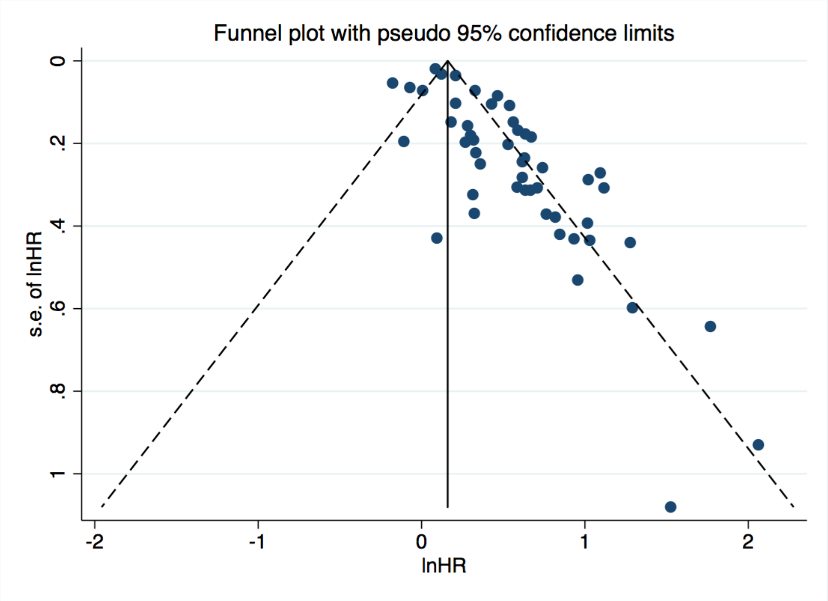

Fig.1B

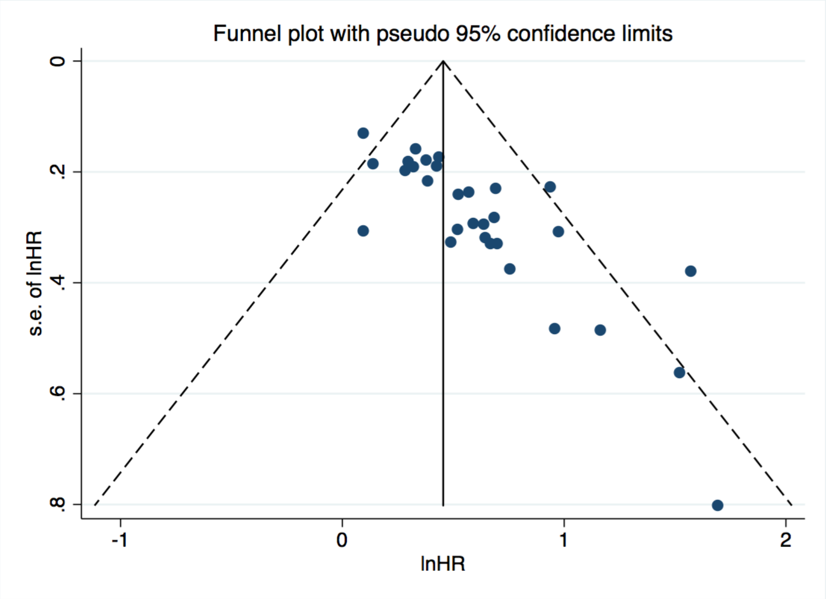

Fig. 1 Publication Bias-Funnel plot for OS set (A) and for DFS/PFS/RFS (B). The funnel plot represents the log HR (on the X axis) against its standard error (on the Y axis) for each individual study (represented by one circle). The vertical line represents the combined effect size, with the diagonal lines representing the expected 95% confidence interval for a given standard error.

Fig.2A

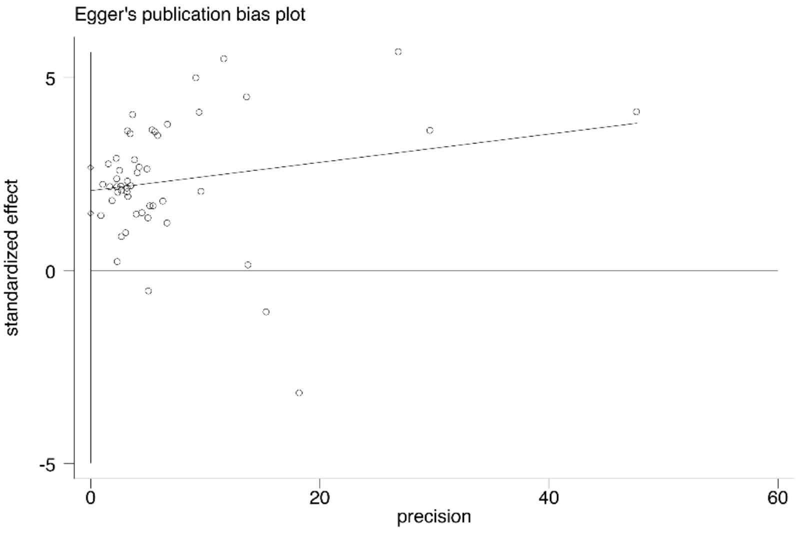

Fig.2B

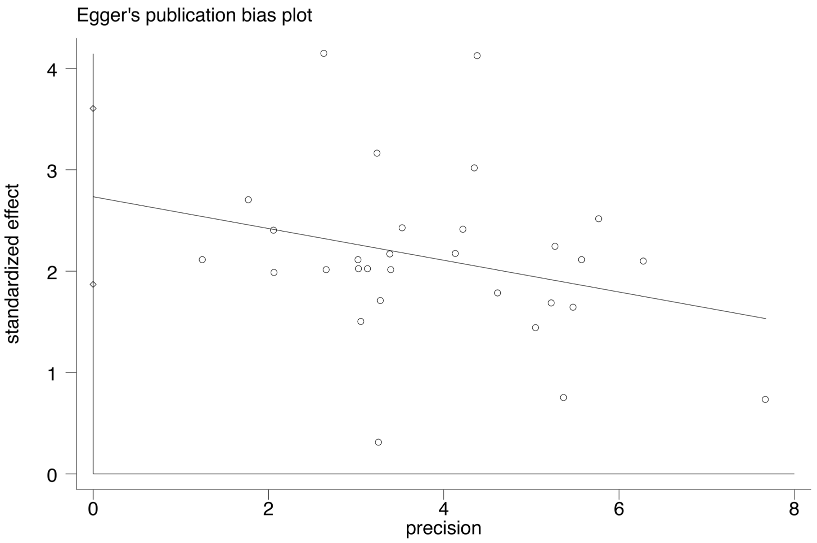

Fig. 2 Publication Bias-Egger's funnel plot for OS set (A) and for DFS/PFS/RFS (B).

Fig.3A

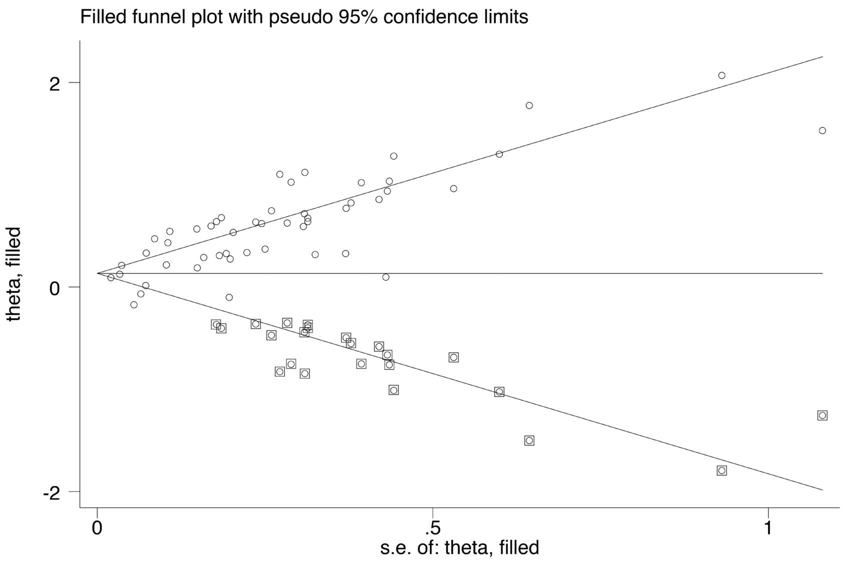

Fig.3B

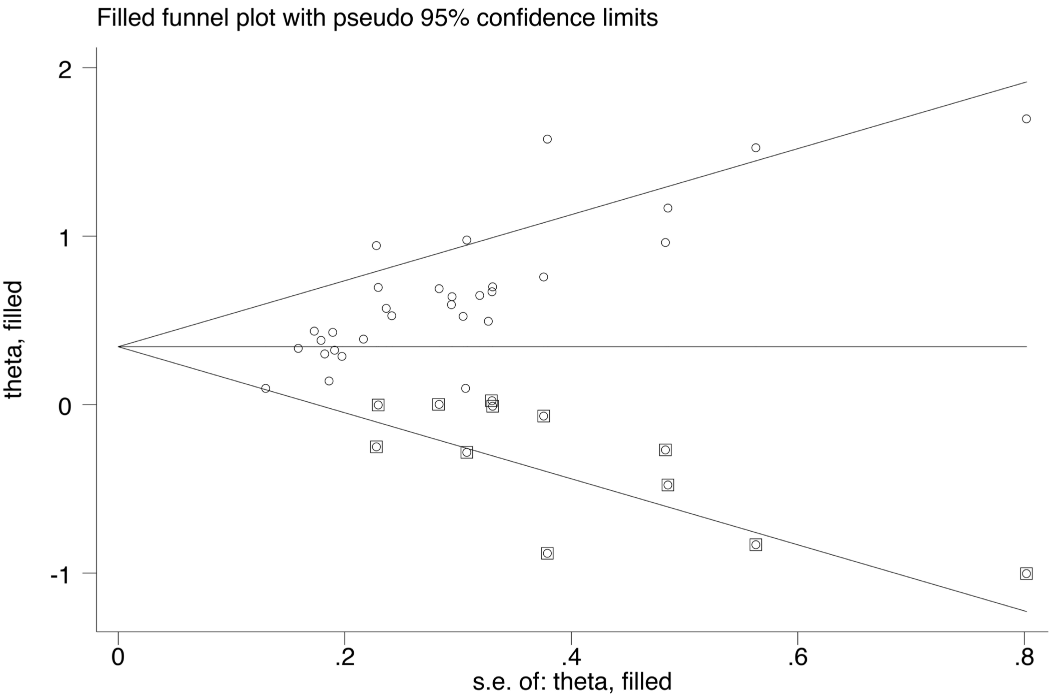

Fig. 3 Adjustment for publication bias among OS set (A) and DFS/PFS/RFS (B) by "Trim and Fill" method.

Supplementary Table. 1 Search Strategies

Search included: PUBMED, EMBASE: search date was from the inception through April 2018

1) Pubmed search strategy

1. "Neoplasms"[Mesh]
2. cancer [Title/Abstract]
3. tumor [Title/Abstract]
4. tumour [Title/Abstract]
5. carcinoma[Title/Abstract]
6. neoplas* [Title/Abstract]
7. malignan*[Title/Abstract]
8. 1 OR 2 OR 3 OR 4 OR 5 OR 6 OR 7
9. "red blood cell distribution width"[MeSH Terms]
10. "red blood cell distribution width "[All Fields]
11. RDW [All Fields]
12. 9 OR 10 OR 11
13. "Survival"[Mesh]
14. "Mortality"[Mesh]
15. "Prognosis"[Mesh]
16. Prognos*[Title/Abstract]
17. outcome*[Title/Abstract]
18. survival[Title/Abstract]
19. mortality[Title/Abstract]
20. predict*[Title/Abstract]

21. 11 OR 12 OR 13 OR 14 OR 15 OR 16 OR 17 OR 18 OR 19 OR 20
22. 8 AND 12 AND 21

2) Embase search strategy

1. 'neoplasm'/exp
2. cancer:ab,ti
3. tumor:ab,ti
4. tumour:ab,ti
5. carcinoma:ab,ti
6. neoplas* :ab,ti
7. malignan*:ab,ti
8. 1 OR 2 OR 3 OR 4 OR 5 OR 6 OR 7
9. red blood cell distribution width'/exp
10. red blood cell distribution width
11. red blood cell distribution width: ab, ti
12. RDW: ab, ti
13. 9 OR 10 OR 11 OR 12
14. 'prognosis'/exp
15. 'survival'/exp
16. 'mortality'/exp
17. prognos*: ab, ti
18. outcome*: ab, ti
19. survival: ab, ti
20. treatment: ab, ti
21. mortality': ab, ti
22. recurren*: ab, ti
23. predict*: ab, ti

24. 14 OR 15 OR 16 OR 17 OR 18 OR 19 OR 20 OR 21 OR 22 OR 23
25. 8 AND 13 AND 24

Supplementary Table. 2 Subgroup analyses of the associations between RDW and DFSPFS/RFS in cancer.

Stratified analyses	No. of patie	No. of studies	Model	Pooled HR (95%CI)	P value	P_D value	Heterogeneity	
							I^2	P_H value
**Tumor type**						<0.001		
Hematologic malignancies	1069	8	Fixed	2.077 (1.644-2.625)	<0.001		9.8%	0.354
MM	552	4	Fixed	1.891 (1.398-2.556)	0.001		40.0%	0.172
DLBCL	242	2	Fixed	2.639 (1.585-4.392)	<0.001		0.0%	0.979
UGI cancer	1293	5	Fixed	1.496 (1.232-1.817)	0.303		0.0%	0.800
HCC	422	2	Fixed	2.104 (1.577-2.807)	<0.001		0.0%	0.516
NSCLC	1241	3	Random	1.418 (1.017-1.976)	0.007		63.9%	0.063
Colorectal carcinoma	753	2	Fixed	1.636 (1.211-2.211)	<0.001		0.0%	0.481
UTUC	420	1*	Fixed	1.520 (1.067-2.166)	<0.001		0.0%	0.789
**Stage**						<0.001		
Mix stage	6293	23	Fixed	1.594 (1.460-1.740)	<0.001		32.6%	0.051
Early stage	964	2	Random	2.192 (0.593-8.109)	0.240		67.8%	0.078
Advanced Stage	54	1	non	non	non		non	non
**Age**						<0.001		
≤ 60	3685	12	Random	1.762 (1.427-2.176)	<0.001		62.1%	0.001
> 60	2552	13	Fixed	1.666 (1.439-1.928)	<0.001		0.0%	0.662
**Gender distribution**						<0.001		
Female dominant	1575	6	Fixed	1.856 (1.462-2.355)	<0.001		49.6%	0.077
Balanced	2505	10	Fixed	1.642 (1.419-1.901)	<0.001		36.9%	0.104
Male dominant	2328	10	Fixed	1.608 (1.400-1.846)	<0.001		0.0%	0.535

**Country**						<0.001		
Eastern	6586	23	Fixed	1.572 (1.437-1.720)	<0.001		39.9%	0.018
Western	764	3	Fixed	1.606 (1.225-2.104)	<0.001		0.0%	0.513
**Cut-off value**						<0.001		
> 15%	78	1	non	non	non		non	non
> 14% and ≤ 15%	2282	10	Fixed	1.546 (1.350-1.771)	<0.001		23.7%	0.218
> 13% and ≤ 14%	2989	9	Random	1.818 (1.474-2.243)	<0.001		54.2%	0.013
≤ 13%	1666	4	Fixed	1.364 (1.092-1.704)	0.006		0.0%	0.700
**Definition of cut-off value**						<0.001		
ROC curve analysis	3656	12	Fixed	1.770 (1.536-2.040)	<0.001		46.5%	0.033
Upper limit	986	6	Fixed	1.647 (1.356-2.001)	<0.001		0.0%	0.731
Median	1219	2	Random	1.515 (0.987-2.326)	0.057		68.5%	0.042
Arbitrary[#]	624	3	Fixed	1.379 (1.081-1.759	0.010		42.5%	0.156
**HR calculation**[‡]						<0.001		
Multivariate	4500	14	Fixed	1.499 (1.347-1.669)	<0.001		46.9%	0.020
Univariate	2850	13	Fixed	1.715 (1.490-1.973)	<0.001		8.2%	0.362

Abbreviations: MM = Multiple Melanomas; DLBCL = Diffuse large B-cell lymphoma; UGI cancer = upper gastrointestinal tract (UGI) cancers (including esophagus cancer, gastric cancer, and small intestine cancer); HCC = hepatocellular carcinoma; NSCLC = non-small cell lung cancer; UTUC = upper tract urothelial carcinoma; OS = overall survival; HR = hazard ratio; CI = confidence interval; P_D = P for subgroup difference; P_H = P for heterogeneity.

*: Cheng et al 2015 separately evaluated the survival outcome in two cohorts, which were derivation cohort and validation cohort.

#: Cut-off value of RDW was based on previous study.
‡: HRs were extracted from multivariate cox proportional hazards models, univariate cox proportional hazards models or survival curve analysis.

Supplementary Table. 3 Methodological characteristics of included studies and quality score.

**No.**	**Authors (Ref.)***	**Representativeness of population**	**Non exposed cohort**	**Ascertainment of exposure**	**Outcome not present at start of study**	**Appropriate confounding measurement and account**	**Sufficient measurement of outcomes**	**Completeness of follow-up**
1	Perlstein et al 2009	0	0	1	1	1	1	1
2	Koma et al 2013	2	1	1	1	2	1	1
3	Abakay et al 2014	1	1	1	1	2	1	1
4	Lee et al 2014	2	1	1	1	2	2	2
5	Riedl et al 2014	1	1	1	1	2	1	0
6	Wang et al 2014	2	1	1	1	2	1	0
7	Warwick et al 2014	2	1	1	1	2	1	2
8	Yao et al 2014	1	1	1	1	2	1	2
9	Chen et al 2015	1	1	1	1	2	1	0
10	Cheng et al 2015	2	1	1	1	2	2	1
11	Iriyama et al 2015	1	1	1	1	1	2	2
12	Periša et al 2015	2	1	1	1	2	2	2
13	Smirne et al 2015	2	1	1	1	2	1	2
14	Wang et al 2015	2	1	1	1	2	1	2
15	Xie et al 2015	1	1	1	1	2	1	1
16	Auezova et al 2016	2	1	1	1	1	1	0
17	Hirahara et al 2016	1	1	1	1	2	1	1

18	Huang et al 2016	2	1	1	1	2	2	0
19	Ichinose et al 2016	1	1	1	1	2	2	1
20	Kos et al 2016	2	1	1	1	1	1	0
21	Liang et al 2016	1	1	1	1	2	1	0
22	Podhorecka et al 2016	2	1	1	1	1	1	0
23	Sun et al 2016	2	1	1	1	1	1	2
24	Uysal et al 2016	1	1	1	1	1	2	1
25	Wan et al 2016	2	1	1	1	2	2	2
26	Zhang et al 2016	2	1	1	1	2	2	2
27	Zhao et al 2016	1	1	1	1	2	2	0
28	Cheng et al 2017	1	1	1	1	1	2	0
29	Howell et al 2017	2	1	1	1	2	1	2
30	Hu et al 2017	2	1	1	1	2	1	2
31	Kara et al 2017	1	1	1	1	2	1	2
32	Kust et al 2017	2	1	1	1	2	1	2
33	Li B et al 2017	2	1	1	1	2	1	0
34	Li Z et al 2017	1	1	1	1	2	1	2
35	Luo et al 2017	2	1	1	1	2	2	2
36	Meng et al 2017	2	1	1	1	1	2	2
37	Sun et al 2017	1	1	1	1	1	1	2
38	Tangthongkum et al 2017	2	1	1	1	2	2	0
39	Wang et al 2017	2	1	1	1	2	1	2
40	Xu et al 2017	2	1	1	1	1	1	0
41	Yazic et al 2017	2	1	1	1	2	1	1

42	Zheng et al 2017	2	1	1	1	1	2	2
43	Zhou et al 2017	2	1	1	1	2	2	2
44	Zhu et al 2017	1	1	1	1	2	2	0
45	Życzkowski et al 2017	2	1	1	1	2	1	2
46	Han et al 2018	1	1	1	1	2	2	2
47	Ma et al 2018	2	1	1	1	1	2	2
48	Zhang et al 2018	1	1	1	1	2	2	2
49	Zhou et al 2018	2	1	1	1	1	2	0

Adequate assessment included 1) representativeness of population: "source population clearly defined" and "study population described" or "study population represents source population or population of interest"; 2) completeness of follow-up: "completeness of follow-up adequate"; 3) non exposed cohort: Drawn from the same community as the exposed cohort; 4) sufficient measurement of outcomes: "outcome measured appropriately"; 5) appropriate confounding measurement and account: "confounders defined and measured" and "confounding accounted for"; and 6) outcome of interest was not present at start of study

*References as described in manuscript

Supplementary Table. 4 Sensitivity analysis using a "one-study removed" model for OS subset.

Sensitivity analysis	Heterogeneity test (I^2)	Pooled HR (95%CI)
All studies	80.0%	1.508 (1.387-1.639)
Excluding Wang et al 2015	78.6%	1.560 (1.420-1.713)

Supplementary Table. 5 Univariate covariate meta-regression analysis for OS subset.

Stratified factors	No. of patients	No. of studies	Coefficients (95%CI)	P value
**Tumor type**				
MM	748	5	0.170 (-0.435, 0.776)	0.568
DLBCL	881	2	0.774 (-0.069, 1.617)	0.071
UGI cancer	3805	6	-0.257 (-0.766, 0.252)	0.309
HCC	1510	5	0.042 (0.468, 0.552)	0.867
NSCLC	2304	4	-0.026 (0.570, 0.518)	0.923
Breast cancer*	2627	3	non	non
Colorectal carcinoma	843	3	0.322 (-0.343, 0.987)	0.329
Gliomas	287	2	-0.030 (-0.625, 0.685)	0.926
UTUC	420	1	0.393 (-0.055, 0.843)	0.215
**Stage**				
Mix stage	16786	33	-0.090 (-0.439, 0.260)	0.609
Early stage	1545	5	0.103 (-0.509, 0.716)	0.736
Advanced Stage*	1416	6	non	non
**Age**				
≤60*	7979	19	non	non
＞60	7992	22	0.031 (-0.198, 0.260)	0.786
**Gender distribution**				
Female dominant*	5059	9	non	non
Balanced	6418	21	0.114 (-0.185, 0.413)	0.446
Male dominant	5325	14	-0.026 (-0.333, 0.279)	0.860
**Country**				
Eastern*	10608	28	non	non
Western	8180	17	-0.157 (-0.366, 0.052)	0.138
**Cut-off value**				

> 15%	3356	6	-0.020 (-0.427, 0.387)	0.921
> 14% and ≤ 15%	7911	21	0.031 (-0.309, 0.371)	0.855
> 13% and ≤ 14%	3409	11	0.189 (-0.183, 0.560)	0.311
≤13%*	1982	5	non	non
**Definition of cut-off value**				
ROC curve analysis	6276	22	0.003 (-0.307, 0.300)	0.982
Upper limit	3558	11	-0.111 (-0.424, 0.202)	0.478
Median	2357	3	-0.204 (-0.615, 0.207)	0.323
4th quartile*	2757	3	non	non
Arbitrary	922	5	-0.139 (-0.558, 0.281)	0.509
**HR calculation**				
Multivariate*	13572	28	non	non
Univariate	4275	17	0.037 (-0.193, 0.268)	0.748

*: This variable was left out of the metareg command in STATA version 14.0 (STATA, College Station, TX). The effect size associated with this variable is labeled _cons in the primary output.

Supplementary Table. 6 Publication bias assessment with different tests for OS and DFS/PFS/RFS.

Publication bias	Begg's *P* value	Egger's *P* value	T&F(Fill) method analysis		Model
			Before	After	
OS subset	0.116	<0.001	1.508 (1.387-1.639)	1.230 (1.132-1.335)	Random
DFS/PFS/RFS subset	<0.001	<0.001	1.576 (1.447-1.716)	1.410 (1.305-1.524)	Fixed

Abbreviations: CI= confidence interval; Fill=number of studies added by trim and fill method; het= heterogeneity; HR=hazard ratio; T&F=result of trimmed and filled analysis, using assumption of random effects.
